# Establishing a hyperacute stroke service during the COVID-19 pandemic: our institution’s one year experience

**DOI:** 10.1186/s12883-023-03102-z

**Published:** 2023-02-15

**Authors:** Anna Misya’il Abdul Rashid, Mohamad Syafeeq Faeez Md Noh, Abdul Hanif Khan Yusof Khan, Wei Chao Loh, Janudin Baharin, Azliza Ibrahim, Fadhilah Hani Ishak, Aminuddin Sardi, Ahmad Firdaus Hanapai, Nur Afiqah Mohamad, Liyana Najwa Inche Mat, Fan Kee Hoo, Wan Aliaa Wan Sulaiman, Hamidon Basri

**Affiliations:** 1 Department of Neurology, Faculty of Medicine and Health Sciences, Universiti Putra Malaysia, 43400 Serdang, Selangor Malaysia; 2 Institut Penyelidikan Penuaan Malaysia (MyAgeing™), Universiti Putra Malaysia, 43400 Serdang, Selangor Malaysia; 3 Department of Radiology, Faculty of Medicine and Health Sciences, Universiti Putra Malaysia, 43400 Serdang, Selangor Malaysia; 4 Department of Neurology, Universiti Putra Malaysia Teaching Hospital, 43400 Serdang, Selangor Malaysia; 5 Center for Foundation Studies, Foundation in Science, Lincoln University College, 47301 Petaling Jaya, Selangor Malaysia

**Keywords:** Hyperacute stroke service, COVID-19, Pandemic, Intravenous thrombolysis, Mechanical thrombectomy

## Abstract

**Background and aims:**

The corononavirus 2019 (COVID-19) pandemic resulted in modifications in the workflow and redistribution of human resources, causing challenges in setting up of an acute stroke service. We would like to share our preliminary outcome amid this pandemic, to determine if the implementation of COVID-19 standard operating procedures (SOPs) affected the delivery of our hyperacute stroke service.

**Methods:**

We retrospectively analyzed one-year data from our stroke registry that began with the establishment of our hyperacute stroke service at Universiti Putra Malaysia Teaching Hospital from April 2020 until May 2021.

**Results:**

Setting up acute stroke services during the pandemic with constrained manpower and implementation of COVID-19 SOPs, was challenging. There was a significant dip of stroke admission from April to June 2020 due to the Movement Control Order (MCO) implemented by the government to curb the spread of COVID-19. However, the numbers of stroke admission steadily rose approaching 2021, after the implementation of recovery MCO. We managed to treat 75 patients with hyperacute stroke interventions i.e. intravenous thrombolysis (IVT), mechanical thrombectomy (MT) or both. Despite implementing COVID-19 SOPs and using magnetic resonance imaging (MRI) as our first line acute stroke imaging modality, clinical outcomes in our cohort were encouraging; almost 40% of patients who underwent hyperacute stroke treatment had early neurological recovery (ENR), and only 33% of patients had early neurological stability (ENS). In addition, we were able to maintain our door-to-imaging (DTI) and door-to-needle (DTN) time in line with international recommendations.

**Conclusions:**

Our data reflects that COVID-19 SOPs did not deter successful delivery of hyperacute stroke services in our center. However, bigger and multi center studies are required to support our findings.

## Introduction

Malaysia is a country in South East Asia, with a total area of 330,345 km^2^ and a population of 32.78 million people. It consist of Peninsular Malaysia and East Malaysia, that is separated by the South China Sea. Stroke is the third leading cause of death in Malaysia, with and incidence of 47, 911 cases, 19,928 deaths, and 512,726 DALYs lost due to stroke in 2019, before the strike on the pandemic [1]. The NINDS and ECAS III trials provided substantial evidence on the efficacy of intravenous thrombolysis (IVT) in acute ischemic stroke (AIS) and since 2008, delivery of IVT is the international standard treatment of care for AIS presenting within 4.5 h [2, 3]. More recent researches in 2015 showed the efficacy of mechanical thrombectomy (MT) in patients with large vessel occlusion (LVO) that is reflected in improved functional outcome at 90 days, with no added risk of bleeding [4, 5].

However, these treatments are required to be delivered within a certain window period of time. This poses as a challenge in our country due to its unique geographical circumstance. The main hindrance is the separation of the main peninsular from the East Malaysia that is located in the Borneo Island. Thus delivering hyperacute stroke treatment is not an easy ordeal, especially in reaching rural ares in Bornoe Island. Hence, hyperacute stroke treatment delivery has been suboptimal in our country, which led the authorities to vigorously improve and promote the development of a stroke network to curb this problem.

Unfortunately, in December 2019, the emergence of a pneumonia of unknown etiology was first reported in Wuhan, China. The etiology was attributed to a novel severe acute respiratory distress syndrome coronavirus (SARS-CoV-2), which was later named COVID-19 [6]. By the end of January 2020, the infection reached East Malaysia through Chinese tourists. However, this first wave was successfully contained, and all infected patients made full recovery. The second wave was a national state of emergency due to its rapid spread through a religious (tabligh) cluster [7, 8]. In April 2020, the Malaysian COVID-19 task force reported a total cumulative case of 6002 people, with 1758 active infections [9].

Despite the peak of this pandemic, we started our hyperacute stroke services at Universiti Putra Malaysia Teaching Hospital in April 2020. The unprecedented pandemic meant that modifications had to be made to the workflow and delivery of our hyperacute stroke service to ensure the safety of both patients and healthcare workers without compromising the quality of stroke care and treatment delivery. It was a challenging pursuit, but the results were encouraging. We wish to share our one-year experience and preliminary outcome during this global pandemic.

## Methods

### Study population

This was a retrospective, single-centre study performed at Universiti Putra Malaysia Teaching Hospital, a tertiary stroke center delivering hyperacute stroke treatment to AIS patients in Malaysia. We included all patients that presented with AIS, whom were referred to us via the Regional Emergency Stroke Quick (RESQ) Response Unit from April 2020 until May 2021 that received hyperacute stroke treatment which consists of IVT-only, MT-only or both IVT and MT (IVT + MT). Patients who received best medical therapy (BMT) were excluded.

### Workflow of RESQ network

We utilized the hub-and-spoke design, in which our hospital serves a main stroke center (hub), offering a full array of hyperacute stroke treatment, in-house neurologists and advanced imaging available round the clock complemented by several smaller hospitals (spoke) with limited services in the region of Klang Valley [10]. This referral system is named the Regional Emergency Stroke Quick (RESQ) Resposne Unit Network, a regional collaboration between Neurologists, Emergency Physicians, Physicians, Radiologists and Interventional Radiologists from private and government health facilities to identify stroke patients who are candidates for hyperacute stroke treatment. The Klang Valley is an urban conglomeration densely populated and centred in the federal territories of Kuala Lumpur and Putrajaya in the state of Selangor in Malaysia, with 7.5 million residents [11].

**Fig. 1:**
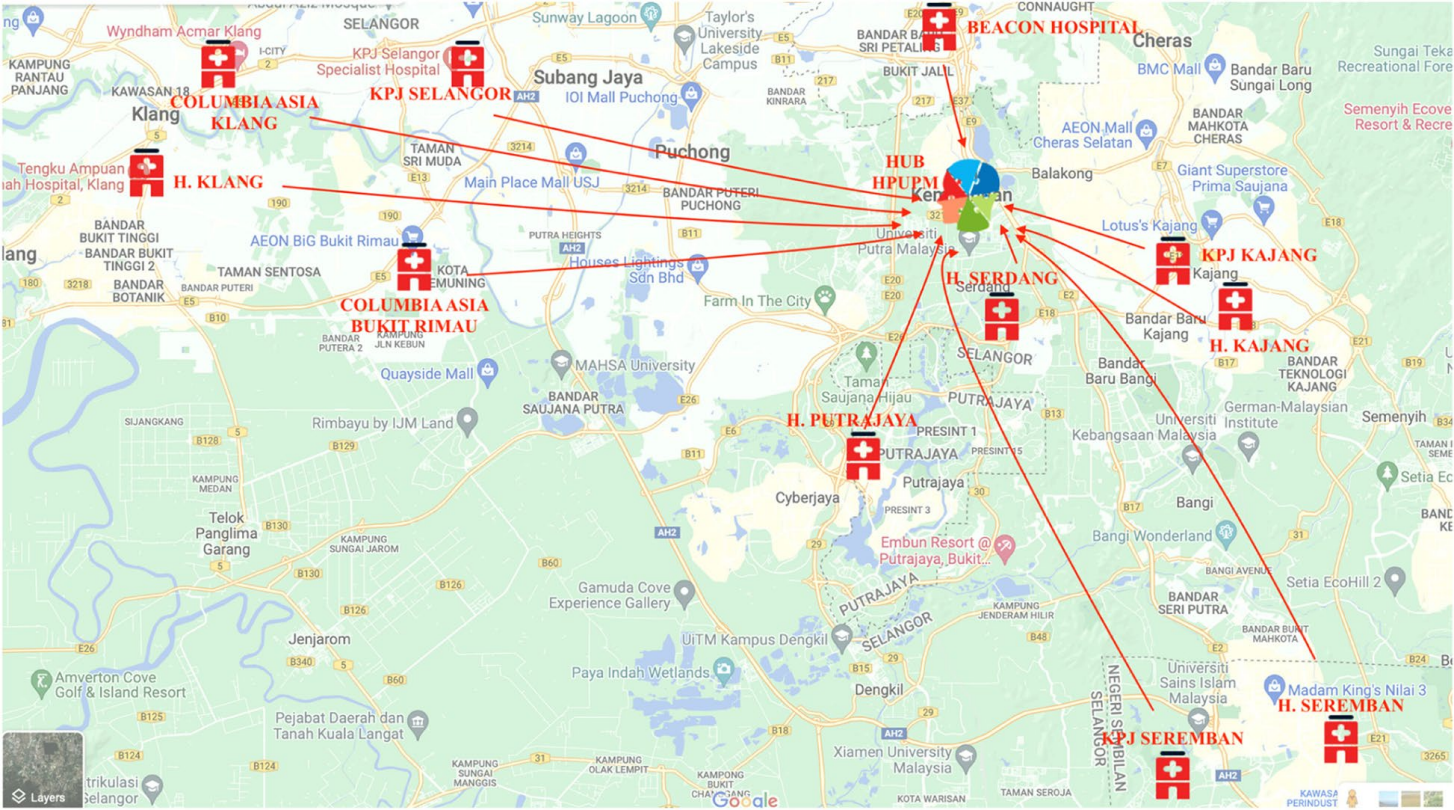
RESQ Network hub-and-spoke map of the Klang Valley region

When patients with stroke symptoms present to the spoke hospitals (Fig. 1), they are assessed for eligibility for hyperacute stroke treatment by Emergency Physicians or Physicians. Most of the spoke hospitals are not equipped with thrombolysis drugs and thus will not be able to administer them prior to transfer. Further more, these hospitals lack advanced imaging such as mangetic resonance imaging (MRI) and computed tomography angiography (CTA), due to lack of adequately trained staffs. Hence, when suitable patients are identified, the stroke code is promptly activated via the RESQ network and patients are transported to our center (hub) for assessment, advanced imaging and delivery of hyperacute stroke treatment. As most of the patients are within vicinity of the hub hospital, patients are able to arrive within 20 to 25 min of transfer. The virtual nature of the RESQ network made it an ideal means of communication and referral between hospitals, especially during the pandemic when limited and controlled movement of patients is necessary to curb the spread of the virus.

### First line imaging

Our center advocates MRI-first policy for the imaging of hyperacute stroke. Although many centers utilize computed tomography (CT), early infarct signs on CT can be subtle and advanced information such as infarct core and volume cannot be determined from CT alone. Thus, we have adopted the Putra Acute Stroke Protocol, where the first three MRI sequences are completed within 8 min, consisting of diffusion-weighted imaging (DWI), fluid attenuation inversion recovery (FLAIR) and magnetic resonance angiography (MRA) [12]. Using data from these three sequences is adequate to determine eligibility for IVT, followed by delivery of IVT in the MRI suite. Our MRI is available around the clock, which included after office hours, weekends and public holidays. An oncall interventional radiologist (IR) is available to interpret MRI images and is also actively involved in the discussion of treatment delivery with the Neurology team. Similarly, allied health workers such as radiographers, staff nurses and medical assistant also have a dedicated stroke team if the angiography suite is required for MT.

### Amendments due to COVID-19 restrictions

**Fig. 2:**
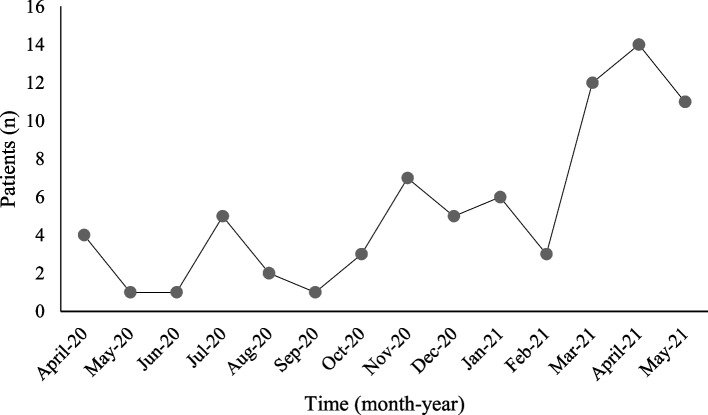
Number of stroke code activation from April 2020 to May 2021

The beginning and peak of the COVID-19 pandemic coincided with the start of our stroke service. As such, the first and second waves of COVID-19 in Malaysia that began in late January to the end of April showed a dip in the number of stroke cases (Fig. 2), as patients were apprehensive about going to the hospital for fear of contracting the virus. As such certain SOPs were implemented to ease patients flow in the emergency department. This included screening for symptoms of COVID-19, temperature and doing the Rapid Test Antigen for COVID-19. Health care workers were also advised to wear personal protective equipment (PPE) during patient encounters, and the number of individuals exposed to the patient is reduced, to limit contact. Although there were concerns at first that these additional SOPs would delay treatment delivery time, our data shows otherwise. Fortunately, our country has dedicated special hospitals and centers for the treatment fo COVID-19, which explained that we did not encounter any COVID-19 positive patients in the first year.

### Measures and definitions

The main objective of this study was to determine if the COVID-19 SOPs implemented affected the delivery of our hyperacute stroke service. Therefore, we sought to obtain demographic information, date and duration of hospitalization for each patient which included age, gender, risk factors and prior strokes. Stroke subtypes were classified by the OXFORD criteria to determine localisation. As delivery of hyperacute stroke service is largely time dependant, door-to-imaging (DTI) time, door-to-needle (DTN) time and door-to-puncture (DTP) is an important measure in this study. We believe that DTI, DTN and DTP time reflects the delivery of our stroke service best, thus decided to analyze these data to see if the COVID-19 SOPs caused delays and subsequently affected the outcome of patients with AIS.

Patient outcome includes clinical findings of the National Health of Institute Stroke Scale (NIHSS) and modified Rankin Scale (mRS) on arrival, discharge, and three months. We also looked into the occurrence of early neurological recovery (ENR); defined as the improvement of the NIHSS by four or more within 24 h, early neurological deterioration (END); which is defined as a worsening of NIHSS of four or more points within 24 h, and early neurological stability (ENS) is defined as a similar NIHSS score within 24 h [13]. Besides that, we also looked at the length of hospital stay after hyperacute stroke treatment.

Safety outcomes include the need for decompressive craniectomy and mortality, including the prevalence of intracranial bleed (ICB) at 24 h, both symptomatic and asymptomatic. Symptomatic ICB is defined by the Heidelberg Bleeding Classification of parenchymatous hemorrhage type 2 (PH2), where the occurrence of the hematoma occupies 30% or more of infarcted tissue with obvious mass effect and above [14].

### Data collection and analysis

The data were extracted from the hospital electronic medical records of patients with AIS using a stroke registry case report form. The medical officers that were responsible for extacting the data were well trained to use the electronic device and they are also trained in the delivery of hyperacute stroke treatment, clinical evaluation and follow up. Data were analyzed on an intention-to-treat basis using SPSS Version 27. Numerical variables (primary outcomes) were checked for normality distribution, and appropriate measures of central dispersion were used to describe the data. Thus, DTI, DTN and DTP were presented as mean (standard deviation [SD]). The categorical variables were presented as frequencies and percentages. This study was approved by Ethics Committee for Research Involving Human Subjects in University Putra Malaysia (Ref: JKEUPM-2022–670).

## Results

**Fig. 3:**
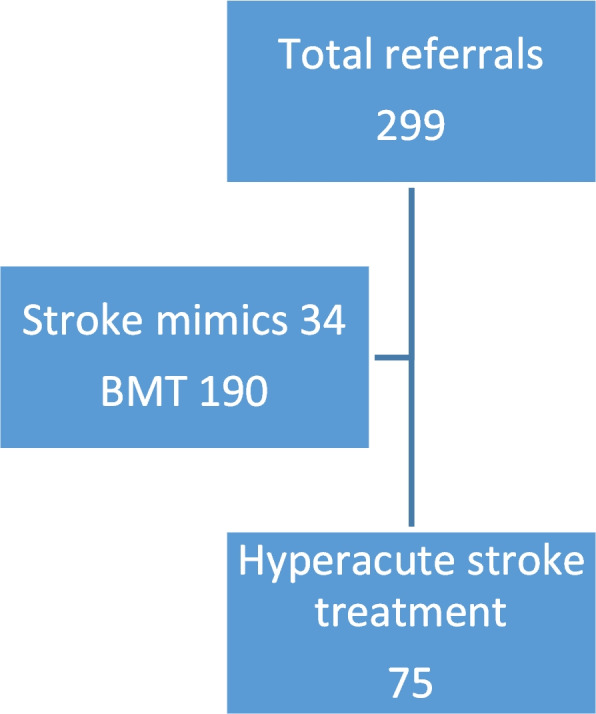
Total number of patients seen in the emergency department. *BMT best medical therapy

From April 2020 to May 2021, we received a total of 299 stroke case referrals from the RESQ network, of which 75 patients underwent hyperacute stroke treatment that includes IVT-only, MT-only or both IVT and MT (IVT + MT). Table 1 shows the demographic profiles of all the patients that underwent hyperacute stroke treatment. One hundred ninety patients received best medical therapy, the scope of which is beyond this manuscript (Fig. 3). Thirty four cases were stroke mimics, thus a total of 224 cases were excluded from the analysis as the outcomes of these patients were not affected by hyperacute stroke treatment.

**Table 1:** Socio-demographic profile of patients undergoing hyperacute stroke treatment

	Treatment group, n (%)			
Parameters	IVT	MT	IVT + MT	Overall
Total	48	11	16	75
Age	57.8 ± 12.4	63.7 ± 13.3	68.0 ± 13.1	60.8 ± 13.2
Gender				
Male	38 (79.2)	4 (36.4)	10 (62.5)	52 (69.3)
Female	10 (20.8)	7 (63.6)	6 (37.5)	23 (30.7)
Co-morbidities				
Diabetes	31 (24.6)	3 (11.1)	7 (19.4)	41 (21.7)
Hypertension	32 (25.4)	9 (33.3)	10 (27.8)	51 (26.9)
Dyslipidemia	26 (20.6)	5 (18.5)	8 (22.2)	39 (20.6)
Smoking	13 (10.3)	3 (11.1)	2 (5.6)	18 (9.5)
Atrial fibrillation	5 (3.9)	1 (3.7)	6 (16.7)	12 (6.3)
CKD	3 (2.4)	1 (3.7)	1 (2.8)	5 (2.6)
IHD	12 (9.5)	3 (11.1)	1 (2.8)	16 (8.5)
Previous stroke/TIA	4 (3.2)	2 (7.4)	1 (2.8)	7 (3.7)
OXFORD classification				
TACI	12 (25.0)	9 (81.8)	13 (81.3)	34 (45.3)
PACI	15 (31.3)	1 (9.1)	2 (12.5)	18 (24.0)
LACI	18 (37.5)	0 (0)	0 (0)	18 (24.0)
POCI	3 (6.3)	1 (9.1)	1 (6.7)	5 (6.7)

*IVT* Intravenous thrombolysis, *MT* Mechanical thrombectomy, *IVT* + *MT* Intravenous thrombolysis and mechanical thrombectomy, *CKD* Chronic kidney disease, *IHD* Ischemic heart disease, *TIA* Transient ischemic attack, *TACI* Total anterior circulation infarct, *PACI* Partial anterior circulation infarct, *LACI* Lacunar infarct and *POCI* Posterior circulation infarct

Overall, the mean age of patients was 60 years old and the majority of the patients are male (69.3%). Most of the patients have comorbidities of hypertension (26.9%), diabetes (21.7%) and dyslipidemia (20.6%). The majority of our patients had anterior circulation infarcts, with only 6.7% having posterior circulation infarcts (Table 1).

Despite the SOPs and additional workflow due to COVID-19 restrictions, we were able to maintain the DTI and DTN, in line with international recommendations (Table 2). Overall the median DTI was 17 min (IQR 13–25), achieving goal DTI of less than 20 min. The median DTN was 46 min (IQR 32.3–60.0), achieving the goal DTN of less than 60 min. The overall median DTP in this cohort was 139 min (IQR 110–152) and did not achieve the intended outcome due to other logistics reasons, including financial clearance and family consent.

**Table 2:** Door-to-imaging, door-to-needle and door-to-puncture time

	Treatment group			
	IVT	MT	IVT + MT	Overall
DTI				
Median	18.5 (13.5–25.0)	15 (10–15)	14.5 (11.3–20.8)	17 (13–25)
Mean	20.2 ± 10.6	20.6 ± 12.2	17.6 ± 7.9	19.7 ± 10.3
DTN				
Median	48.5 (36.5–64.5)	NA	39.5 (24.0–54.3)	46 (32.3–60.0)
Mean	51.5 ± 26.8	NA	40.8 ± 18.7	48.8 ± 25.3
DTP				
Median	NA	130 (110–153)	139 (102–149.5)	139 (110–152)
Mean	NA	140.6 ± 39.1	127.1 ± 37.0	132.6 ± 37.7

^*^Data reported as Median (*IQR* interquartile range) and Mean ± SD

The clinical findings which included the mean NIHSS and mRS on arrival, during discharge and at three months. We also took into consideration the occurrence of ENR, END and ENS (Table 3). The median NIHSS for the IVT-only group is 10 (IQR 6–14) and is higher in the MT-only group (16 [IQR 5.3–21.3]) and MT + IVT group (18 [IQR 6.5–18]). The IVT-only group has the best improvement of median NIHSS score at discharge, four (IQR 1–8), and at three months, NIHSS one (IQR 0–3). The MT-only group did not show improvement in median NIHSS at discharge (17.5 [IQR 3.8–19.8), but at three months, the median NIHSS score was 5.5 (IQR 0–12.8). Similarly, in the IVT + MT group, the immediate neurological recovery was not seen at discharge with a median NIHSS of nine (IQR 2–20), but at three months, the median NIHSS was 6.5 (IQR 3–12.3).

**Table 3:** Clinical outcomes of patients receiving hyperacute stroke treatment

	Treatment group			
Outcomes	IVT	MT	IVT + MT	Overall
Clinical assessment on arrival				
NIHSS (Median)	10 (6–14)	16.5 (5.3–21.3)	18 (11–21)	11 (6.5–18.0)
NIHSS (Mean)	10.4 ± 5.8	14.3 ± 8.0	16.8 ± 5.4	12.2 ± 6.5
mRS (Median)	0	0	0	0
mRS (Mean)	0.3 ± 0.8	0.1 ± 0.3	0.5 ± 1.2	0.3 ± 0.9
Clinical assessment on discharge				
NIHSS (Median)	4 (1–8)	17.5 (3.8–19.8)	9 (2–20)	4.5 (2.0–12.8)
NIHSS (Mean)	5.5 ± 5.9	14.3 ± 9.4	10.7 ± 7.8	7.5 ± 7.3
mRS (Median)	3 (1–4)	5 (3–6)	5 (3–6)	4 (1–5)
mRS (Mean)	2.8 ± 1.7	4.3 ± 1.8	4.3 ± 1.7	3.3 ± 1.9
Clinical assessment at 3 months				
NIHSS (Median)	1 (0–3)	5.5 (0.0–12.8)	6.5 (3.0–12.3)	2.0 (0.0–5.3)
NIHSS (Mean)	2.3 ± 4.1	6.3 ± 6.2	7.6 ± 6.4	3.6 ± 5.1
mRS (Median)	1 (0–4)	5 (1–6)	5.5 (3–6)	2 (1–6)
mRS (Mean)	2.0 ± 2.2	3.8 ± 2.4	4.5 ± 1.8	2.8 ± 2.4
ENR	25 (52.1)	1 (9.1)	4 (25.0)	30 (40.0)
ENS	17 (35.4)	4 (36.4)	4 (25.0)	25 (33.3)
END	6 (12.5)	6 (54.5)	8 (50.0)	20 (26.7)
Length of stay (days)	8.8 ± 7.52	9.1 ± 4.91	13.0 ± 9.20	9.8 ± 7.69

*NIHSS* National institute of health stroke scale, *mRS* modified rankin scale, *ENR* Early neurological recovery, *ENS* Early neurological stability, *END* Early neurological deterioration

Consequently, the occurrence of ENR was highest in the IVT-only group with almost 52.1%, followed by 25% in the IVT + MT group and 9% in MT only group. END, on the other hand was more prevalent in the IVT + MT and MT-only groups (50% and 54.5%, respectively) and was lowest in the IVT-only group (12.5%).

The mean length of stay was lowest for the IVT-only group, which was eight days (SD ± 7.52). The number of hospitalization days was slightly longer in the MT-only group, 9.1 days (SD ± 4.91) and IVT + MT group, 13 days (SD ± 9.2).

With regards to safety outcomes (Table 4), there was a total of 19 cases of ICB overall; 13 (68.4%) were asymptomatic, and 6 (31.5%) were symptomatic. The highest number of ICB was in the IVT-only group with ten patients, but nine (90%) were asymptomatic, and only one (10%) was symptomatic. The patient underwent decompressive craniectomy and had a good recovery at three months.

**Table 4:** Safety outcomes of patients receiving hyperacute stroke treatment

	Treatment group, n(%)			
	IVT	MT	IVT + MT	Overall
ICB at 24 h				
Asymptmatic	9 (90)	1(25)	3 (60)	13 (68.4)
Symptomatic	1 (10)	3 (75)	2 (40)	6 (31.5)
Mortality	3	3	5	11

*ICB* Intracranial bleed

In the MT-only group, there were four ICBs; three (75%) were symptomatic, but did not undergo decompressive craniectomy due to poor prognosis and eventually passed away due to ICB. One (25%) patient had asymptomatic ICB and did not require surgery. He continued to do well even at three months post-stroke.

In the IVT + MT group, there were five ICB, three (60%) were asymptomatic and two (40%) were symptomatic but did not undergo decompressive craniectomy due to poor prognosis and eventually passed away.

There 11 mortality overall, and the highest was in the IVT + MT group, which consisted of five patients (45%). Two patients died of cerebral edema and mass effect and two patients died due to ICB. All four patients did not receive decompressive craniectomy due to poor prognosis. One patient died due to septic shock.

There were three mortalities in the MT only group, the cause of death being ICB. All three did not undergo decompressive craniectomy due to poor prognosis and passed away during admission. In the IVT only group, three patients died but only one was due to complications of bilateral mid brain stroke while the other two cases were due to cardiogenic shock and septic shock.

## Discussions

The COVID-19 pandemic has affected the delivery of hyperacute stroke services globally, with many countries seeing a decline in stroke code activation and the number of patients presenting with stroke in the emergency department [15, 16]. Many centers reported that the decline in the patient presentation was due to movement restriction orders causing difficulty to reach health care facilities, while others reported reduced stroke code activation and delivery of health care systems due to restriction of beds and utilization of intensive care units for the care of COVID-19 patients [17–19].

As the only center in our region providing comprehensive hyperacute stroke treatment, our center was fortunate to not have restrictions on bed allocations despite the climbing numbers of COVID-19 cases. However, we were affected indirectly as our spoke hospitals struggled to triage possible stroke cases and activation of stroke code, while managing an overwhelming number of COVID-19 patients. This effect was worst during the first MCO implemented in March 2020, that restricted patient movement across borders of counties and states [20].

This was reflected in the number of our stroke code activations, that saw a dip during April to June 2021. The number of cases continues to fluctuate during the upcoming months, as the nation staggered back and forth between conditional movement control order (CMCO) and recovery MCO, which allowed some easing of movement restrictions; and subsequently back to the second MCO in January 2021 (Fig. 2). However, after vaccination was introduced, the severity of COVID-19 is seen to decrease, allowing us to sustain the number of patients via the RESQ network and emergency department.

The pandemic spread has led to workflow modifications in the emergency department. Patients were screened with a questionnaire, and a COVID-19 rapid test was done before full neurological examination and imaging. Health providers are required to don PPE, consisting of N95 masks, face shields, gloves and gowns, before attending patients. Despite our concerns that this might prolong DTI and DTN, our numbers show otherwise, where we achieved a median DTI of 17 min across all groups and DTN of 46 min. This was within international recommendations, which called for DTI of less than 20 min and DTN of less than 60 min.

However, the DTP was affected at an overall median of 139 min (international recommendation of less than 90 min). This phenomenon is echoed worldwide, and some stroke centers also struggle to keep up with DTN and DTP [21, 22]. Velez et al. reported that the prolonged DTP in their center was due to COVID-19 SOPs that required negative pressure room for intubation and the need for PPE and powered air purifying respirator (PAPR) while securing the airway prior to MT [17]. In our center, this was mainly due to financial restrictions. As a semi-government hospital we did not receive full subsidiary for MT. Therefore, a certain amount of money had to be deposited before the beginning of MT, and many families struggled to mobilize emergency funds on such short notice. This was made worse as the government channelled most health care funds to battling COVID-19 infections, and allocations for other diseases were significantly cut.

Fortunately, despite the COVID-19 SOPs, the outcomes of our patients who underwent hyperacute stroke treatment was not greatly affected. This is reflected in the proportion of patients in the overall cohort who achieved ENR was 40% and ENS was 33%, amounting to almost 73% who have benefitted from early treatment. This has signifantly reduced hospitalization days, which in turn decreased inpatient care burden on allied health care workers, especially nurses. This was important in our center as some of the nurses were deployed to COVID-19 centers, in an attempt to control the disease outbreak.

We are aware that the delay in DTP may have affected our outcome negatively. Considering that our DTI were on time, the outcomes would have been better if the DTP were shortened. We observed a higher number of symptomatic ICBs in our MT only and IVT + MT group. However, in both these groups, the median NIHSS score during arrival was higher (16.5 [IQR 5.3–21.3] and 18 [IQR11-21]) as compared to the IVT-only group (10 [IQR 6–14]) which could have contributed to a worse outcome and a bigger size of ICB. Consequently, the infarct volume was larger in these group, thus, complications of hemorrhagic transformation and cerebral oedema was also higher, which contributed to mortalities in both these groups. Of note, all the death in the MT-only group were due to large middle cranial artery (MCA) infarcts. In the IVT + MT group, three out of five deaths were due to large MCA infarcts that evolved into ICBs, and one was due to basilar artery occlusion (BAO), which carries a 90% mortality rate [23].

The limitation of this study is the retrospective nature and that data is extracted from a single center, thus our findings may not be generalized to all stroke centers. As such, making sound recommendations for establishing hyperacute stroke care in times of pandemic is difficult. However, we wish to shed some insight to readers about our struggle and pursuit to deliver hyperacute stroke service, despite the daunting shadow of the COVID-19 pandemic. Although the battle was tough, we were able to treat a considerable number of patients who would have otherwise had long-term neurological sequalae.

## Conclusions

The battle against COVID-19 has been long and ghastly, affecting many fields in the medical fraternity. However, the pursuit providing health care for all acute emergencies besides COVID-19 should not be overlooked. Our data reflects that COVID-19 SOPs did not deter successful delivery of hyperacute stroke services in our center. However, bigger and multi center studies are required to support our findings.

## Data Availability

The datasets supporting the conclusions of this article are included within the article.

## Abbreviations

COVID-19: Coronavirus 2019
SOP: Standard operating procedures
MCO: The movement control order
IVT: Intravenous thrombolysis
MT: Mechanical thrombectomy
ENR: Early neurological recovery
END: Early neurological deterioration
MRI: Magnetic resonance imaging
DTI: Door-to-imaging
DTN: Door-to-needle
SARS-CoV-2: Severe acute respiratory distress syndrome coronavirus
WHO: World health organization
AIS: Acute ischemic stroke
RESQ: Regional emergency stroke quick response unit
CT: Computed tomography
DWI: Diffusion-weighted imaging
FLAIR: Fluid attenuation inversion recovery
MRA: Magnetic resonance angiography
PPE: Personal protective equipment
DTP: Door-to-puncture
NIHSS: National health of institute stroke scale
mRS: Modified rankin scale
ENS: Early neurological stability
ICB: Intracranial bleed
SD: Standard deviation
CMCO: Conditional movement control order
PAPR: Powered air purifying respirator
MCA: Middle cranial artery
BAO: Basilar artery occlusion
LVO: Large vessel occlusion
